# Machine Learning as a Method for Retrieving Pressure Values by Analyzing Spectral Line Parameters: The Hydrochloric Acid Case

**DOI:** 10.1021/acsphyschemau.5c00097

**Published:** 2025-11-05

**Authors:** Alexandre E. Santos, Laiz R. Ventura, Carlos E. Fellows

**Affiliations:** † Departmento de Física, Instituto de Ciências Exatas—ICEx, 28110 Universidade Federal Fluminense, Campus do Aterrado, Volta Redonda, RJ 27213-45, Brazil; ‡ Departamento de Física, 74360 Instituto Tecnológico de Aeronáutica, 12228-900 São José dos Campos, Brazil

**Keywords:** machine learning, hydrochloric acid, line broadening, infrared absorption

## Abstract

This study proposes a noninvasive machine learning approach to infer pressure by analyzing the infrared spectral lines of the HCl molecule. High-resolution spectra were simulated using the HITRAN database across various pressures (15–900 mbar), temperatures (273–373 K), and optical paths (1–10.5 cm). Voigt profile parameters (amplitude, center, height, and Gaussian/Lorentzian widths) were extracted from these spectral lines and used to train six ML models. The ExtraTrees algorithm demonstrated superior performance, achieving an RMSE of 23.95 mbar on synthetic data. Validation with experimental spectra (78–790 mbar, 293 K) revealed strong agreement at lower pressures, with errors below 5% (e.g., 2.62% at 78 mbar). The hybrid methodology, which combines simulated training with experimental validation, circumvents the need for direct sensor exposure to corrosive environments and offers a reliable alternative for pressure retrieval.

## Graphical abstract

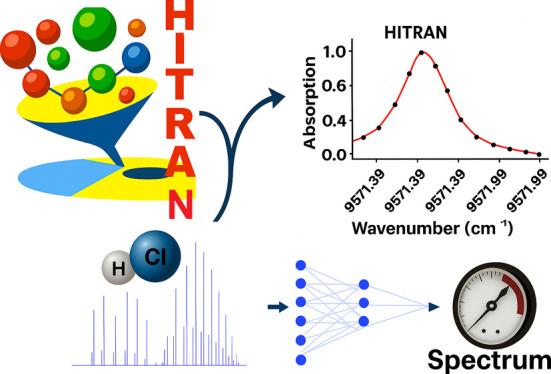

## Introduction

Molecular spectroscopy has been a fundamental tool for characterizing the physicochemical properties of substances and molecules, allowing the analysis of molecular structures and interactions under different environmental conditions. In particular, high-resolution spectra of molecules such as hydrochloric acid (HCl), a species of significant astrochemical and industrial relevance, offer insights into various phenomena, such as broadening and deviations of spectral lines.^1–4^ However, the quantitative extraction of physical parameters from these spectra, such as the pressure of the system, presents intrinsic experimental challenges. In the case of HCl, a highly reactive and corrosive gas, direct pressure measurements are often compromised by the accelerated degradation of conventional sensors, limiting the reliability and durability of detectors under prolonged operating conditions.^5,6^ This technical limitation reinforces the need for alternative, noninvasive approaches to inferring parameters such as pressure, for example, without exposing instruments to irreversible damage, thus mitigating high operating costs. In addition, alternative approaches to retrieving data such as pressure and temperature are of great value in atmospheric measurements.

In this context, the use of machine learning (ML) techniques has emerged as a promising approach to automating and improving spectroscopic analysis. ML has revolutionized several areas of physics, as exemplified by the work of Schleder et al.,^7^ which highlights how ML bridges quantum simulations and data-driven discovery, enabling rapid predictions of electronic structures and thermodynamic stability. Another example is the recent work of Duarte, Nemmen, and Navarro,^8^ that demonstrates ML’s potential in astrophysics by accelerating accretion flow simulations by ×10^4^ compared to traditional methods. In spectroscopy, artificial intelligence, particularly machine learning models, has emerged as a superior alternative for accurately predicting VUV/UV absorption spectra, even outperforming computationally intensive quantum chemical methods as shown by Manh et al.^9^ In addition, ML models such as Voting Regressor have been applied to predict pressure-broadening parameters for exoplanetary atmospheres, achieving 69% accuracy in reproducing experimental data and enabling faster radiative transfer simulations, as demonstrated by Guest, Tennyson, and Yurchenko.^10^

The study of HCl is fundamental not only for terrestrial applications, but also for understanding planetary and interstellar environments.^4^ For example, HCl has been detected in the atmospheres of Earth,^11^ Venus,^12^ and Mars,^13^ where it influences the radiative balance, cloud formation and surface-atmosphere interactions.^14,15^ On Venus, its presence is correlated with the release of volcanic gases and sulfur cycles, while on Mars, it serves as a marker for underground Cl reservoirs and transient atmospheric chemistry.^16^ Beyond our solar system, HCl is a key probe for deciphering the molecular complexity of star-forming regions and evolved stellar envelopes. In this work, we present a new methodology for estimating the pressure of gaseous systems from high-resolution HCl spectra, using ML models trained with Voigt profile fitting parameters.

To overcome the experimental challenges associated with HCl corrosivity, the models were initially trained and optimized on simulated spectra based on the HITRAN database, which provides high-precision molecular parameters for controlled conditions. Subsequently, the method was validated on real experimental spectra, demonstrating robust generalization of the models between synthetic and experimental data. The experimental spectra used to validate the method were recorded at a temperature of 293 K and at five different pressures: 78, 145, 200, 398, and 790 mbar. Each spectral line was modeled as a Voigt function, whose parameters (amplitude, Gaussian and Lorentzian widths, and center) served as input for ML algorithms. The results show a robust convergence between real and estimated values, with percentage differences of less than 5% at lower pressures (e.g., 78 mbar) and acceptable accuracy even at higher pressure ranges (790 mbar), where challenges such as line overlap and broadening effects are critical. This hybrid approach, combining HITRAN simulations with experimental data, not only validates the efficacy of the model, but also offers a safe path for analyzing reactive gases, minimizing the prolonged exposure of detectors to corrosive species.

## Experimental Procedure

A Bruker IFS 125HR Fourier-transform spectrometer (with approximately 2 m of optical path length) equipped with an LN_2_ cooled InSb detector and a Si/Ca coated beam splitter was used to record the infrared spectra of the 2–0 band of the HCl molecule. In order to reduce the noise, a total of 50 interferograms were coadded with a resolution of 0.050 cm^–1^. At a temperature of 293 K, the high-resolution absorption spectra were conducted at five different pressures: 78, 145, 200, 398, and 790 mbar. A 100 mm Pyrex absorption cell with quartz windows was used for the measurements.

Considering that hydrogen chloride consists of two predominant isotopic forms, H^35^Cl and H^37^Cl, with a natural abundance ratio of approximately 3.1267:1,^17–19^ both species were simultaneously detected and analyzed in this study. A total of 25 rovibrational transitions for each isotope, spanning the *P*(12) to *R*(12) range, were experimentally recorded and examined in each employed pressure. Through the use of the OPUS^20^ software package, the experimental wavenumber of the spectral lines was determined from the observed spectrum. More details about the experiment can be found in the work of Santos et al.^4^

## Methodology

The development of accurate machine learning models for spectroscopic applications requires complete data sets that reliably represent the physicochemical variations of the phenomenon under study. However, the experimental generation of spectra under a variety of physical conditions faces significant obstacles: (i) the complexity and cost of experimental systems that allow controlled pressure variations; (ii) the extended time required to systematically acquire measurements in different experimental configurations; and (iii) the particular challenge of exploring different combinations of operating parameters, such as different optical path lengths, in different pressure and temperature ranges. These practical constraints typically limit the amount and variety of experimental data available, thereby compromising the ability to train sophisticated predictive models with the necessary generalization. The methodology developed and applied in this work is presented in Figure 1, with each step described in the following subsections.

**Figure 1:**
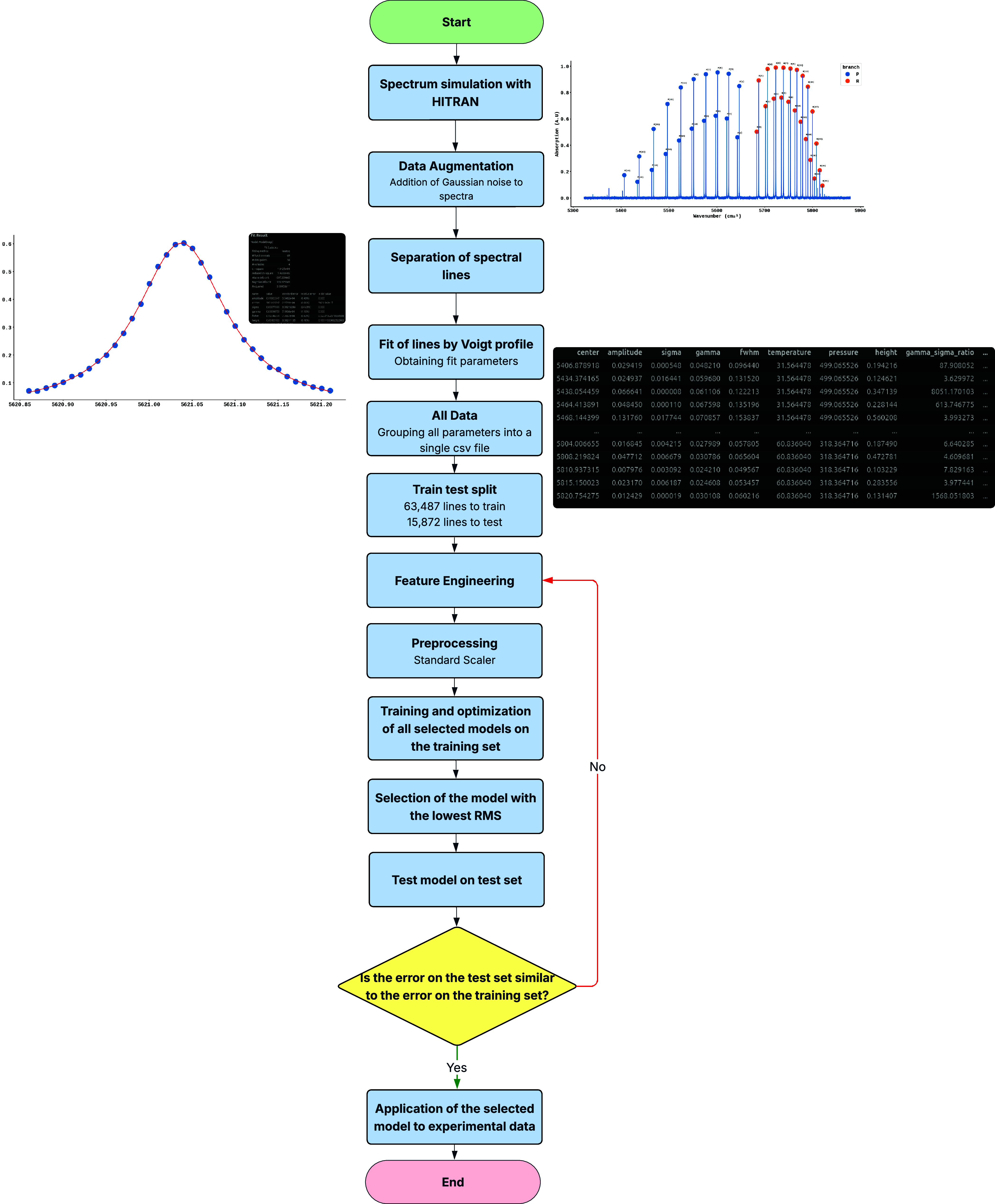
Schematic representation of the methodology developed and applied in the present work.

### Input Data

Due to the experimental limitations exposed, it was obtained five spectra under different pressures: 78, 145, 200, 398, and 790 mbar. All spectra were recorded at a temperature of 293 K. To overcome this limitation, we used the HITRAN database to simulate the absorption spectra of hydrochloric acid under various pressures, temperatures, and optical path conditions. The spectra were simulated and organized into five different blocks, each containing 500 simulations, for a total of 2500 spectra generated. For each simulation, it was necessary to establish the pressure, temperature, and optical path. They were then sampled independently, following a uniform distribution. The intervals defined were: temperature from 273 to 373 K, pressure from 15 mbar to 900 mbar, and optical path from 1 to 10.5 cm.

The data used as input for training and testing the models were exclusively simulated spectra generated from the HITRAN database. This approach allowed the generation of a comprehensive set of 2500 spectra systematically covering different pressure, temperature, and optical path conditions. The experimental spectra available were reserved exclusively for the final validation stage, thus ensuring an independent assessment of the models’ performance under real conditions. Figure 2 shows a comparison of the experimental and simulated spectra for part of the *R* branch of the 2–0 vibrational band of the HCl molecule, recorded at a temperature of 293 K and a pressure of 78 mbar. The upper trace corresponds to the high-resolution experimental spectrum, while the lower trace represents the simulation derived from the HITRAN database at the same temperature and pressure. As can be seen, there is a clear agreement between the measured and simulated spectral profiles. The isotopic composition of HCl is clearly resolved, with transitions to H^35^Cl (more intense lines) and H^37^Cl (less intense lines) identifiable.

**Figure 2:**
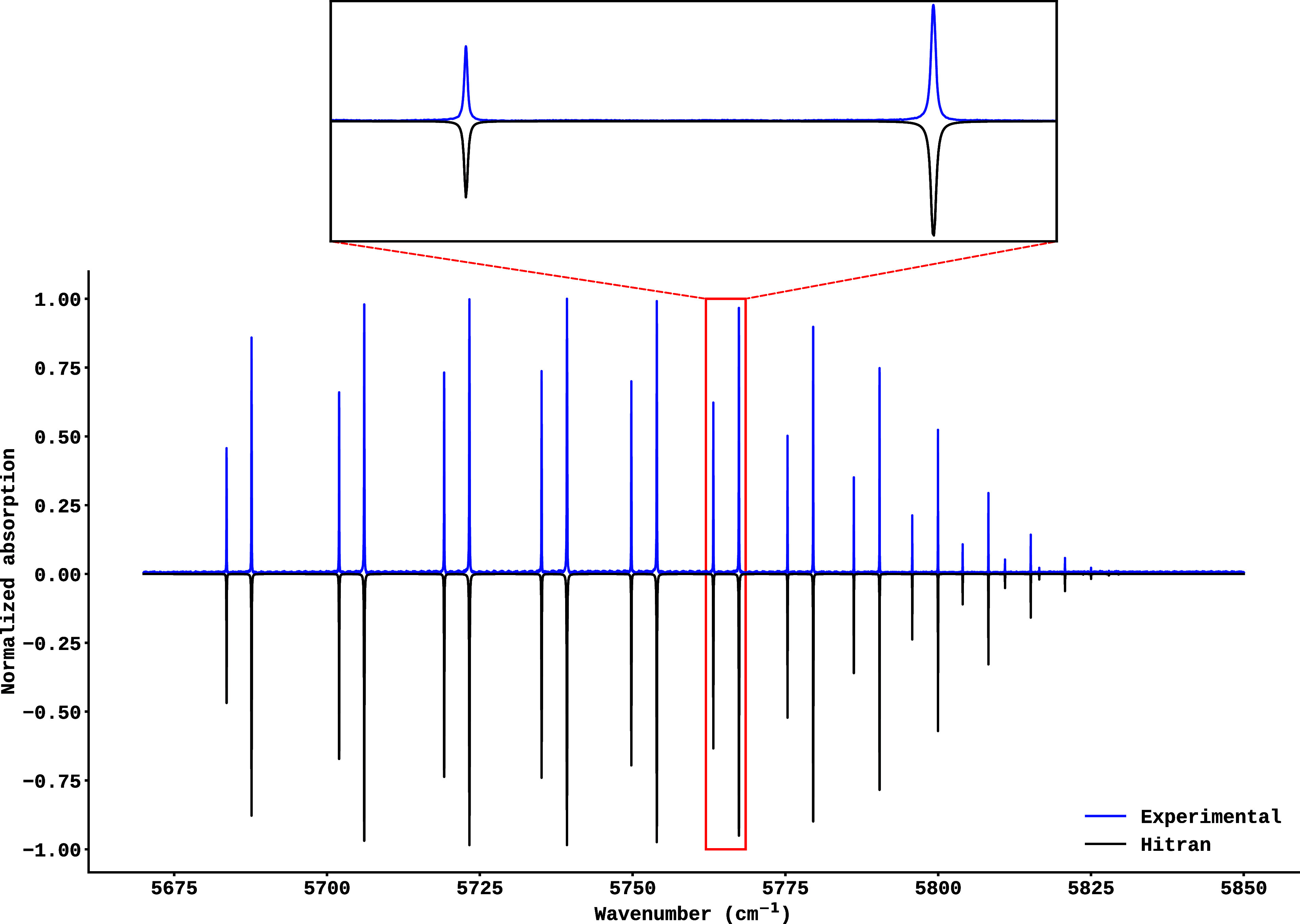
Part of *R* branch in the high-resolution spectrum of the 2–0 vibrational band of HCl molecule, recorded at *T* = 293 K and a pressure of 78 mbar. The experimental (upper trace) and HITRAN simulation (lower trace) spectra are compared. The isotopic composition of HCl is resolved, highlighting distinct transitions for H^35^Cl (more intense lines) and H^37^Cl (less intense lines).

### Feature Extraction from Spectra

The simulated spectra went through a preprocessing stage, in which each spectral line was analyzed individually to extract the features relevant to training the models. To do this, we fitted a Voigt profile to each line using the Python lmfit package.^21^ The Voigt profile can be expressed using eqs 1 and 2,

f(x;A,μ,σ,γ)=A·Re[w(z)]σ2π 1

where

$$\begin{array}{rl} z & =\frac{x-\mu +i\gamma }{\sigma \sqrt{2\pi }},\\ w(z) & =e^{-z^{2}}\mathrm{erfc}(-iz) \end{array}$$ 2

From this fit, six physical parameters were obtained: amplitude *A*, center *x* (line position), σ (Gaussian broadening), γ (Lorentzian broadening), full width at half height (fwhm) and height (maximum peak height). Figure 3 shows the fit of an experimental spectral line (A) and the corresponding fit simulated by HITRAN (B), both performed under the same physical conditions and using the Voigt profile. The residuals of each fit, shown below the respective curves, show fluctuations close to zero, indicating the accuracy and quality of the fit performed. From these fits, the parameters mentioned above were extracted, which were used as inputs for training machine learning models.

**Figure 3:**
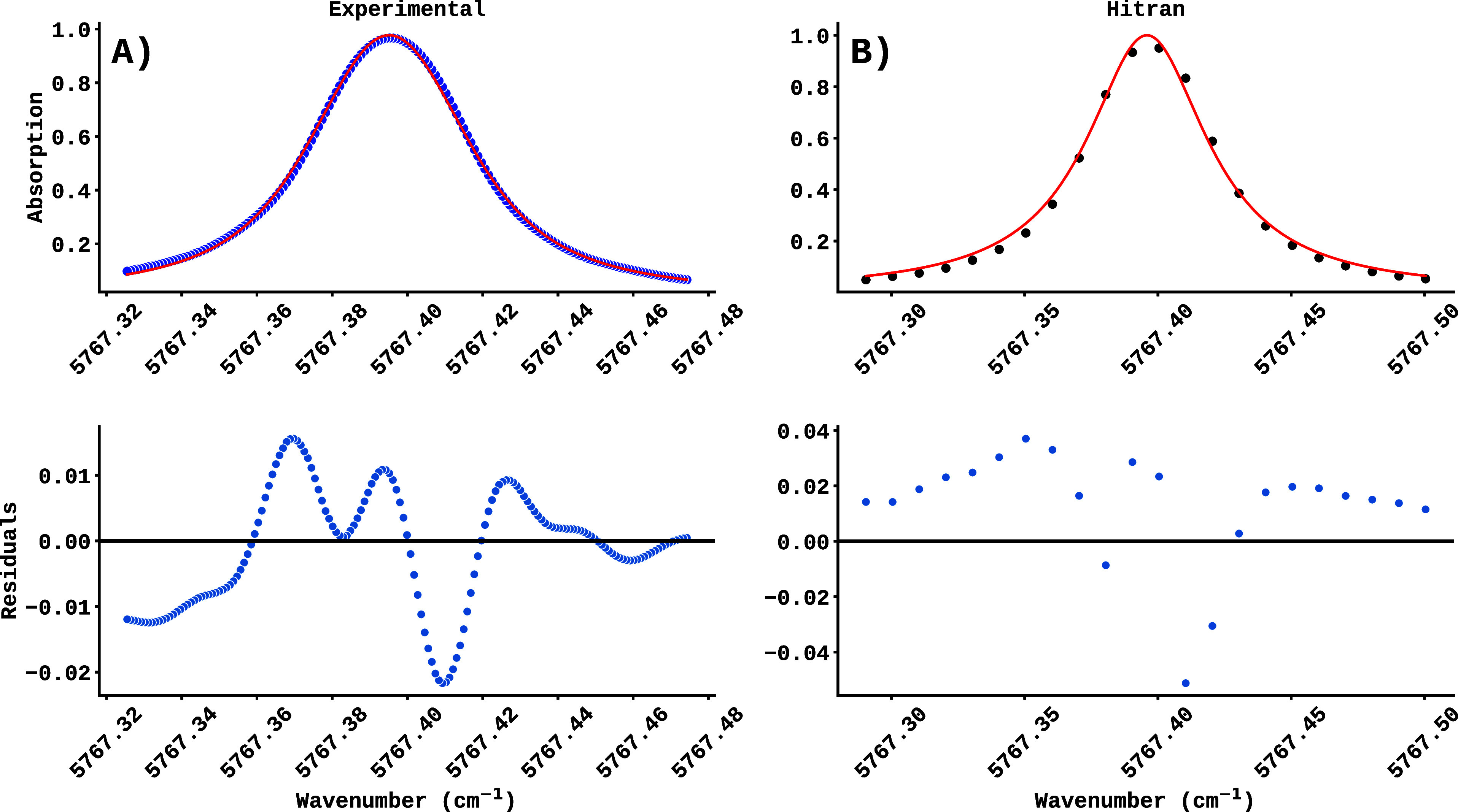
Result of fitting a spectral line using the Voigt function (red curve) is shown for an experimental case (A) and one simulated by HITRAN (B). The residuals of the fits are shown below each graph, with the black line indicating the ideal fit between the data and the model.

In addition to the features obtained directly from fitting the Voigt profile, it was implemented a complementary stage of feature engineering. In this phase, new features were generated through mathematical combinations (e.g., addition, multiplication, ratios) of the original variables, significantly expanding the data representation space and capturing possible nonlinear interactions between the physical parameters. These parameters, derived directly from Voigt’s model, were used as input variables for the machine learning algorithms, ensuring that the physical information intrinsic to the spectra guided the learning process.

Obtaining these features resulted in variables with different scales. To ensure that all the variables made a balanced contribution to the model, the input data was submitted to a preprocessing pipeline using the StandardScaler method from the scikit-learn library.^22^ This technique standardizes each feature individually, centering the data around zero (mean = 0) and adjusting its standard deviation to a unit (σ = 1). This process is essential to prevent variables of greater magnitude from dominating the model.

### Machine Learning Models

After the features extraction, it was selected six machine learning models: ExtraTrees,^23^ Extreme Gradient Boosting (XGB),^24^ Light Gradient Boosting Machine (LGBM),^25^ Random Forest,^26^ K-Nearest Neighbors (KNN)^27^ and Decision Tree.^28^ All the algorithms were implemented using Python’s scikit-learn python package,^22^ with the exception of XGB^29^ and LightGBM,^30^ which, although they have interfaces compatible with the library, belong to independent ecosystems.

K-Nearest Neighbors (KNN) is a similarity-based algorithm that computes distances (such as Euclidean or Manhattan) between data points to identify the *k* nearest neighbors and make predictions by voting (classification) or averaging (regression). In contrast, a Decision Tree is hierarchically structured by decision nodes that apply rules (such as partitioning based on entropy or Gini index) to recursively partition the data into increasingly homogeneous subgroups until reaching leaves (final nodes) that represent the predictions.^31^ Both are basic models, but ensemble techniques combine multiple trees for greater robustness. Random Forest and Extra Trees, for example, are bagging ensembles: they train multiple trees in parallel, each with random subsets of data (bootstrapping) and, in the case of Random Forest, a restricted set of features in each partition. Extra Trees also add randomness to the partitioning criteria. XGBoost and LightGBM (LGBM) follow boosting, training trees sequentially: each new tree focuses on the residuals (errors) of the previous one, gradually minimizing the loss function.^31^

### Train and Test

Each model used was trained independently on each of the simulated blocks using the cross-validation method. Figure 4 shows a comparison of the performance of the six machine learning models employed (ExtraTree, Lgbm, RandomForest, Xgb, DecisionTree and Knn) by means of the Root Mean Square Error (RMSE), evaluating each algorithm on five different samples, each containing 500 spectra. In this graph, the *y*-axis represents the RMSE values, while the *x*-axis identifies the models. For each model, five individual bars correspond to the results obtained on each data sample. The average RMS (Mean RMS), shown above the bars, reflects the average of the errors grouped by model, considering the five samples. In addition, the standard deviation (Std), also shown above the bars, quantifies the variation of the results around this average, highlighting the consistency or dispersion of each algorithm’s performance.

**Figure 4:**
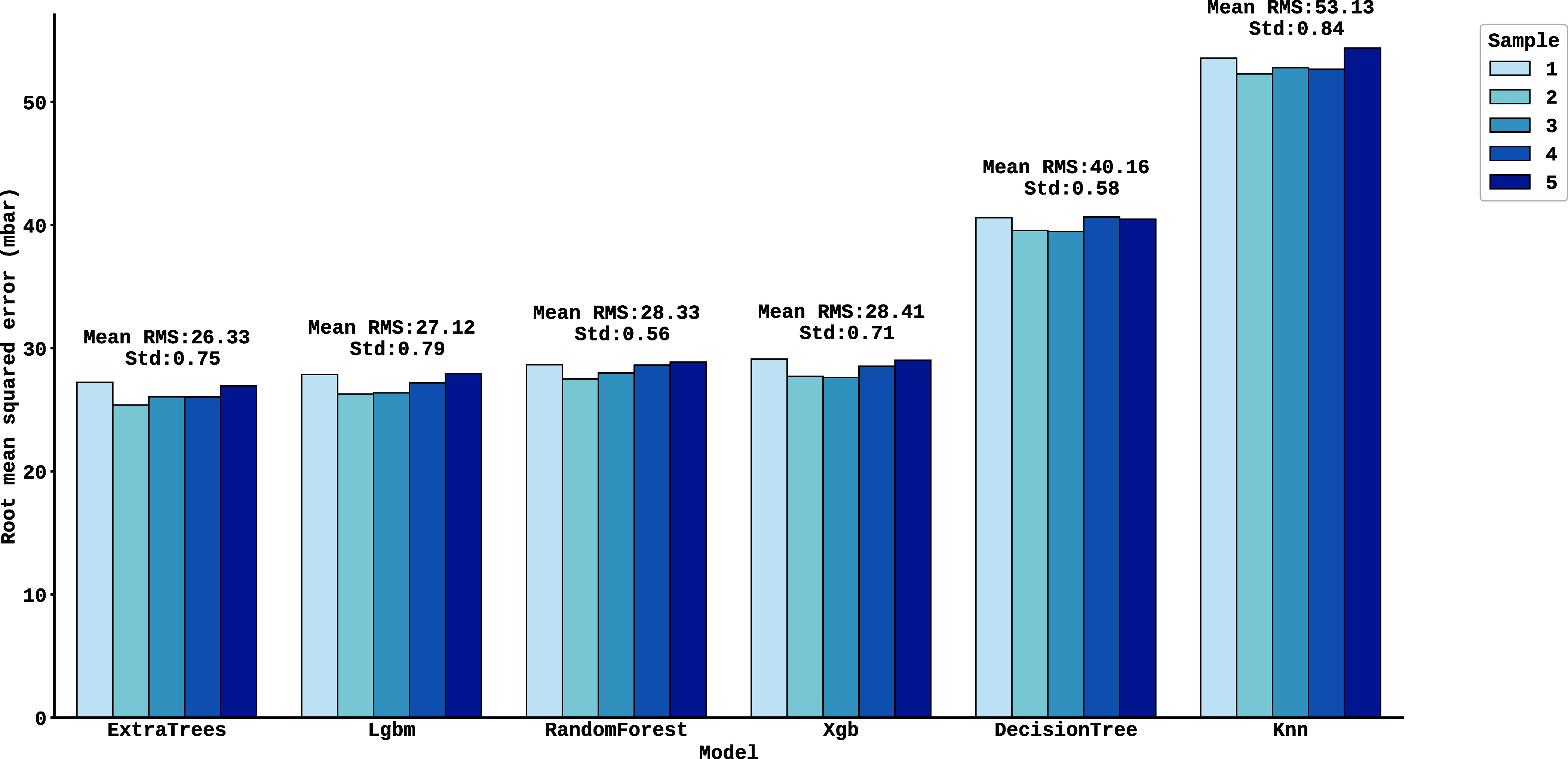
Comparison of the performance of six machine learning models (ExtraTree, Lgbm, RandomForest, Xgb, DecisionTree and Knn) using the Root Mean Square Error (RMSE), evaluating each algorithm on five different samples, each containing 500 spectra.

It can be seen from Figure 4 that the results obtained by all the models in each block show similar performance, which rules out the possibility of random bias in the generation of spectra and confirms that the algorithms effectively learned relevant patterns present in the simulated data. To assess average performance, it was calculated the average error and standard deviation of this error for the predictions, taking into account the five independent samples per model. The ExtraTrees model showed the best performance, with an average RMSE of 26.33 mbar between blocks, followed by LightGBM (RMSE = 27.12 mbar). In contrast, K-Nearest Neighbors (KNN) had the lowest performance, registering a higher RMSE of 53.1 mbar.

Given the consistency observed in the individual samples, the five samples were consolidated into a single data set, called the total set, made up of 2,500 spectra. The features were then extracted and standardized using scikit-learn’s StandardScaler method,^22^ resulting in a final data set with 79,359 processed lines and 57 features per line. Subsequently, the data was divided into training and test sets, following a ratio of 80% for training (63,487 lines) and 20% for testing (15,872 lines). In this way, the data preparation process ensured the reproducibility and robustness of the subsequent training and validation stages.

It is presented in Figure 5 the Root Mean Square Error (RMSE) for each model used, calculated using cross-validation applied to the training set. The results obtained with the total data set showed slightly better performance compared to the individual samples in Figure 4, although the trends and magnitudes of error remained consistent between both approaches. It was observed that the tree-based models (such as LightGBM, ExtraTrees and Random Forest) achieved the lowest errors, with RMSE between 24 mbar and 25 mbar. In contrast, Decision Tree recorded an error of 34 mbar, while K-Nearest Neighbors (KNN) achieved the worst performance, with an RMSE of 41 mbar. This performance hierarchy reinforces the effectiveness of ensemble techniques for the task in question.

**Figure 5:**
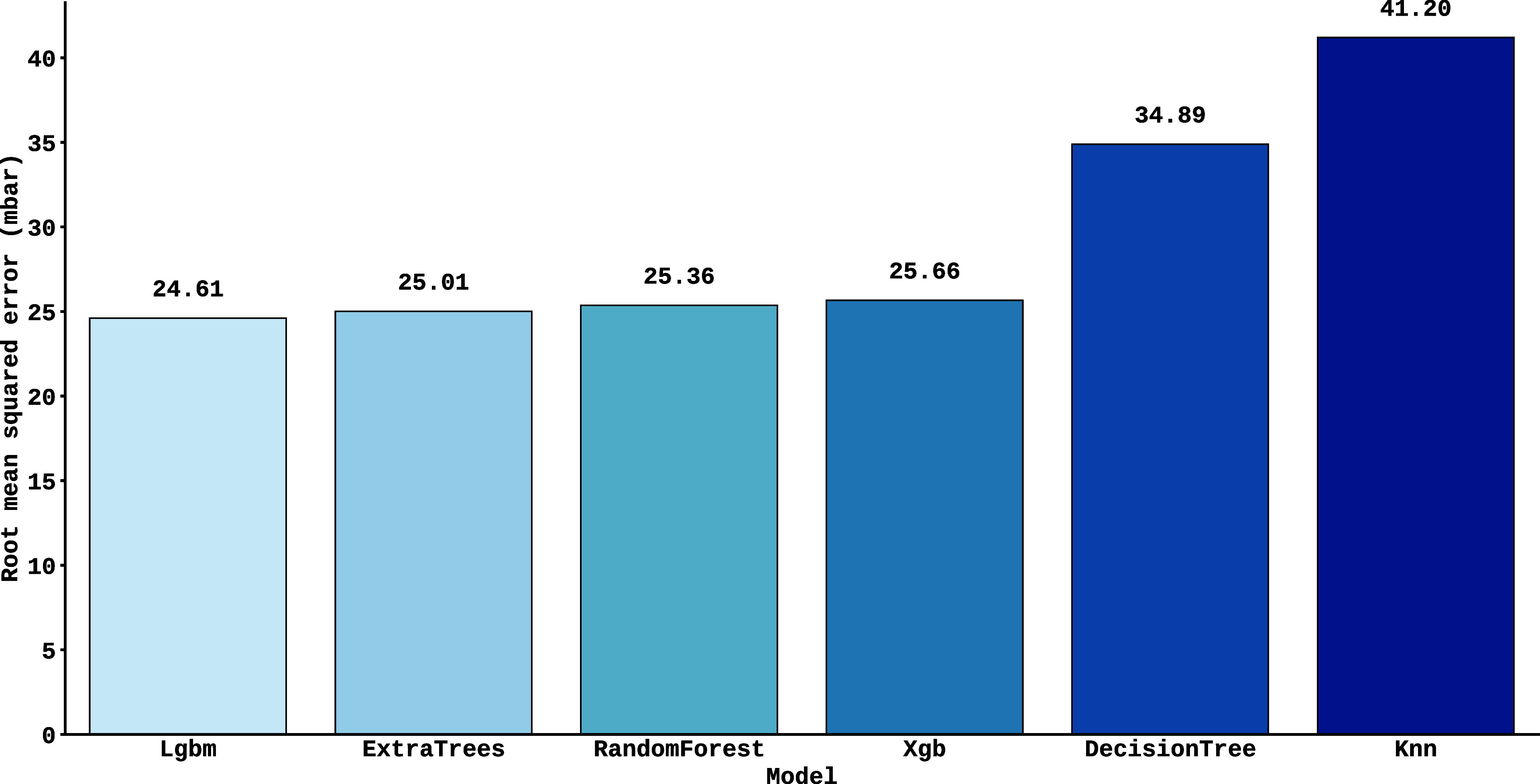
Figure shows the Root Mean Square Error (RMSE) of models trained with 2500 synthetic spectra, with the *y*-axis representing the RMSE values and the *x*-axis the identification of the algorithms. The results show the performance of each model in determining pressure.

In the final training stage, we fine-tuned the models’ hyperparameters in order to further reduce prediction errors. To do this, we employed a Bayesian search strategy using the BayesSearchCV class from the skopt package,^32^ combined with cross-validation, in order to identify optimized configurations. The consolidated results are shown in Figure 6, where the *y*-axis indicates the models, while the *x*-axis represents the Root Mean Square Error (RMSE). For each algorithm, there are two horizontal bars: the light blue corresponds to the RMSE without optimization, and the dark blue to the RMSE after optimization. Next to each pair of bars, a text details three pieces of information: the first is *No* (RMSE without optimization), the second is *Yes* (RMSE with optimization) and the third is Δ (percentage variation). Positive Δ*v*alues indicate an improvement in performance after optimization, while negative values suggest a worsening, making it possible to quickly visualize the impact of adjusting the hyperparameters on each model.

**Figure 6:**
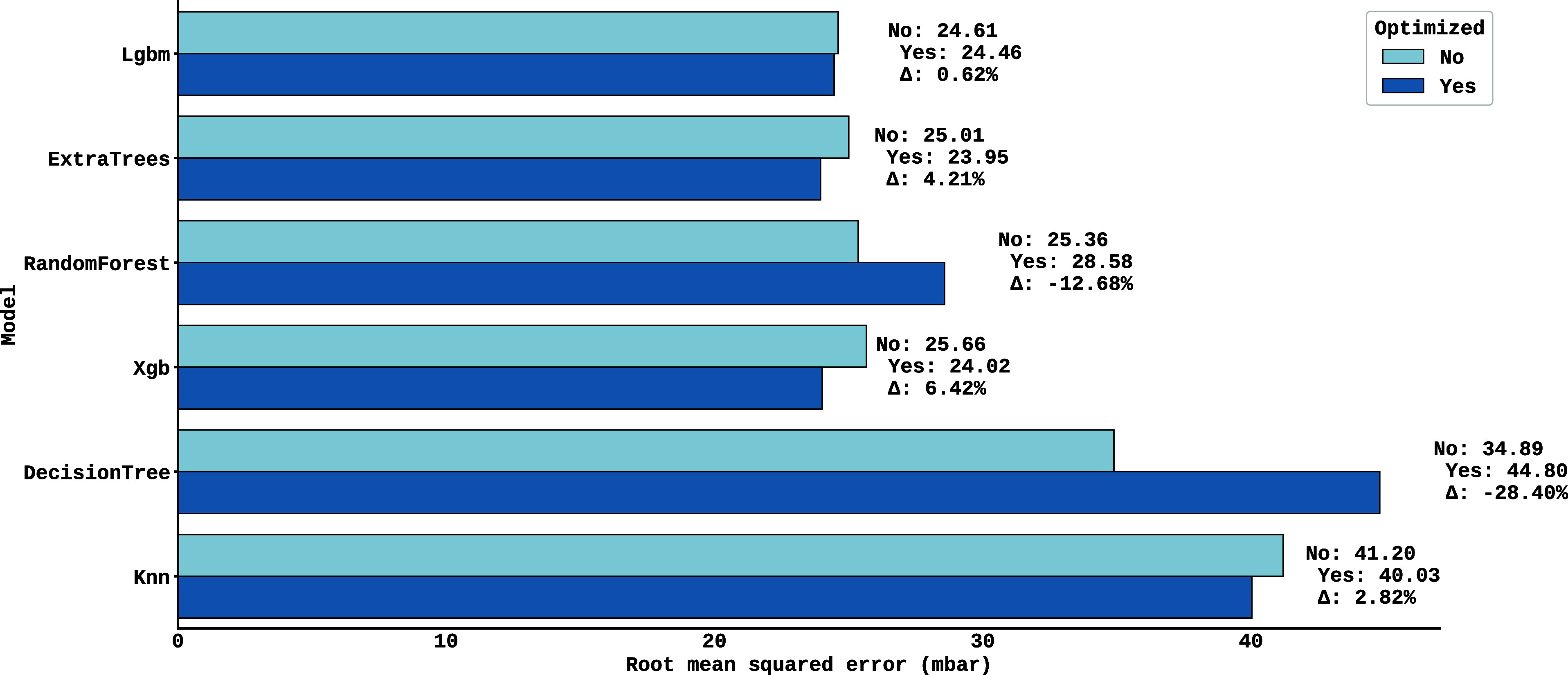
Comparison of performance between models before and after hyperparameter optimization is presented. The *y*-axis indicates the models, while the *x*-axis represents the Root Mean Square Error (RMSE).

It can be observed from Figure 6 significant variations in performance after optimization: while some models showed significant gains (such as XGBoost, which reduced its RMSE from 25 mbar to 24 mbar, an improvement of approximately 6%), others showed worsening, such as Decision Tree, whose RMSE increased from 34 to 44 mbar with the new hyperparameters. The ExtraTrees model remained the model with the lowest error, reaching a final RMSE of 23.95 mbar after optimization. As a result, the optimized parameters for the model were maximum tree depth equal to 17, minimum number of samples for splitting a node equal to 17, and number of estimators equal to 362. The other hyperparameters, such as the impurity criterion, the bootstrap method, and others, remained according to the default settings of the scikit-learn library.

It should be noted, however, that all the ensemble models (LightGBM, Random Forest, XGBoost, and ExtraTrees) showed similar metrics. It is important to note that each optimization process was limited to 100 iterations, which may have restricted the ability of the search algorithms to explore more promising hyperparameter spaces. This limitation probably contributed to suboptimal results in certain cases, such as the increased error in the Decision Tree.

Given the results, the ExtraTrees model with the optimized hyperparameters was trained using the entire training set (63,487 lines). Its generalization capacity was then assessed independently on the test set (15,872 lines), which remained isolated throughout the adjustment and optimization stages.

After processing the test data using the trained model, the resulting pressure estimates are shown in Figure 7. This graph plots the estimated pressures for the synthetic spectral lines (*y*-axis) against the simulated real pressures (*x*-axis), where the dashed black line represents the ideal model, while the blue circles correspond to the spectral lines of the test set. Despite the significant alignment with the ideal curve, there is a progressive increase in the error at higher pressures, evidenced by the gradual divergence of the blue dots from the reference line (black line). This trend is reinforced by the analysis of the residuals shown in Figure 8, which highlights the model’s greater difficulty in predicting accurate values at higher pressure regimes. A pressure-dependent bias is observed, with residues increasing with increasing pressure. The distribution of errors, analyzed by histogram, shows a symmetry close to a normal or Lorentzian profile. To quantify the concentration of the residuals, an interval covering 95% of the data was defined, bounded by the 2.5% (−52.57) and 97.5% (51.29) percentiles, represented by the red lines. The red areas highlight the residues outside this range, while the green area indicates those within the range. Although the range encompasses most of the data, it does not correspond to a statistical confidence interval because the residuals exhibit heteroscedasticity and systematic pressure dependence. This analysis is intended only to illustrate the dispersion of errors across the full range of pressures considered, and to highlight the conditional nature of the observed bias. In addition, the root-mean-square error of the ExtraTrees model in the test set was 23.34 mbar, which is very close to the result obtained with the same metric in the training set (23.95 mbar), indicating consistent performance and the absence of overfitting.

**Figure 7:**
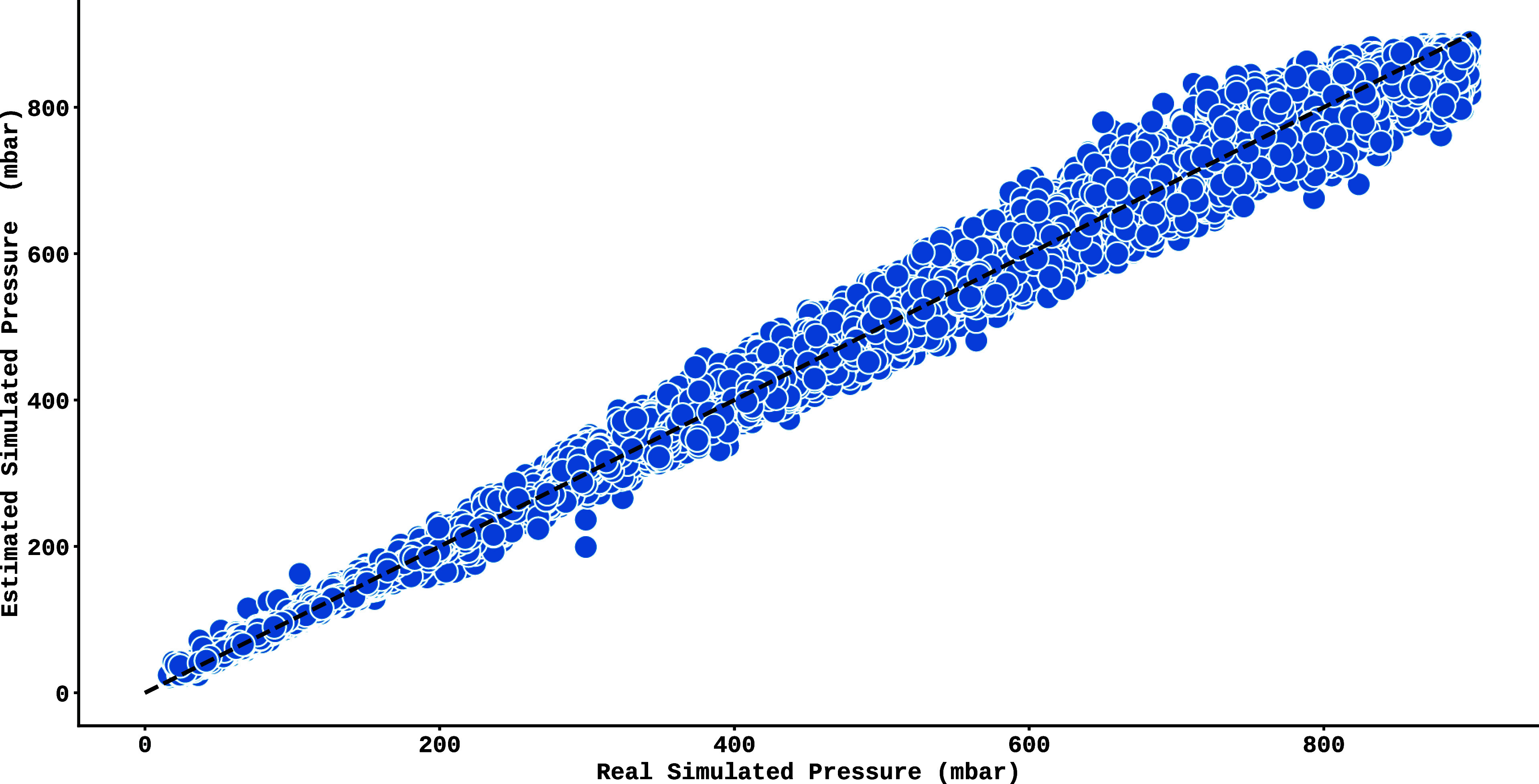
Validation of the Extra-Trees model, trained and tested on synthetic spectra. The *y*-axis indicates the pressure estimated by the model, while the *x*-axis shows the actual pressure values.

**Figure 8:**
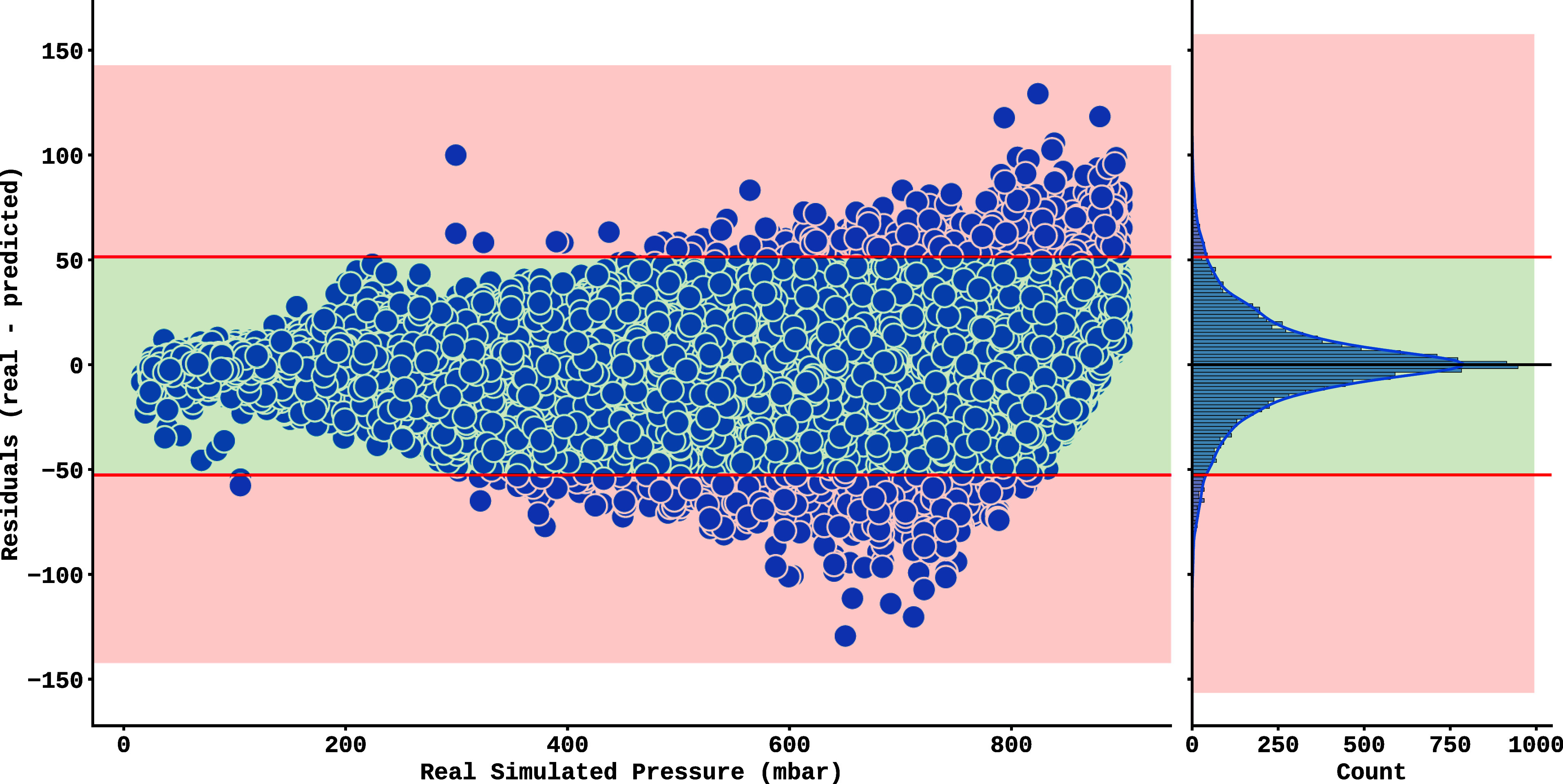
Residuals (difference between actual and predicted values) of the Extra-Trees model applied to synthetic test data, plotted against the actual simulated pressure values on the horizontal axis. The histogram on the left shows a symmetrical distribution of the residuals, with a slight shift in the mean to 0.28.

## Results and Discussion

According to the schematic representation of the methodology developed and applied in this work, shown in Figure 1, it can be seen that, after selecting the model with the lowest RMS, this model is then applied to the experimental data. In the present case, the model selected was the Extra-Trees, and its results applied to the experimental data are shown in Table 1, which is organized into four columns: real pressure (experimentally measured values), estimated pressure (model predictions), absolute difference (|real – estimated|), and percentage difference $\left(\frac{\left|\mathrm{real}-\mathrm{estimated}\right|}{\mathrm{real}}\times 100\right)$ . Each line corresponds to an analyzed spectrum, allowing a direct comparison of the accuracy of the predictions with the experimental data. The absolute difference quantifies the error in pressure units, while the percentage difference represents this error relative to the magnitude of the actual value, making it easier to assess the relative accuracy of the model in different pressure ranges.

**Table 1:** Results of the Extra-Trees Model Applied to Real Spectra, Organized into Four Columns: Real Pressure, Estimated Pressure, Absolute Difference, and Percentage Difference^a^

real pressure (mbar)	estimated pressure (mbar)	absolute difference (mbar)	percentual difference (%)
78	75.96	2.04	2.62
145	151.39	6.39	4.40
200	204.37	4.37	2.18
398	347.97	50.03	12.57
790	558.55	231.45	29.30

a Each line corresponds to an analyzed spectrum, allowing a direct comparison of the accuracy of the predictions with the experimental data.

Using the data presented in Table 1, it is shown in Figure 9 the validation of the Extra-Trees model on experimental spectra, comparing the pressure estimated by the algorithm (*y*-axis) with the real values measured by physical detectors (*x*-axis). Each point corresponds to a real spectrum, whose estimated pressure is calculated as the average of the predicted pressures for each of the approximately 40 spectral lines that make up the spectrum. The black dashed line (*y* = *x*) represents the ideal relationship between prediction and experimental data, and the proximity of the points to this line indicates the accuracy of the model under experimental conditions. The dispersion of the points around the ideal line makes it possible to visually assess the consistency and possible trends of the model in estimating pressure in real situations. It was observed that the model has increasing difficulty in estimating pressure values as the magnitude increases. For pressures up to 200 mbar, the absolute errors were between 2 and 6 mbar, corresponding to a relative error of 2–4%. However, at higher pressures, such as 398 mbar, the absolute error increased to 50 mbar (12%), and at 790 mbar the error was 230 mbar (29%), showing a significant evolution of the error with increasing pressure. In addition, a tendency for the model to underestimate the real values in regions of high pressure was observed, a behavior that is inconsistent with the patterns observed in the training and test data, whose observed behavior for the errors was symmetrical around the real value. This bias suggests that the model may be oversimplifying nonlinear relationships or suffering from a lack of representative data at the extremes of the distribution. The increase in relative error (from 4 to 29%) indicates the need for improvements, such as the inclusion of new variables or possibly the development of a new model for higher pressures.

**Figure 9:**
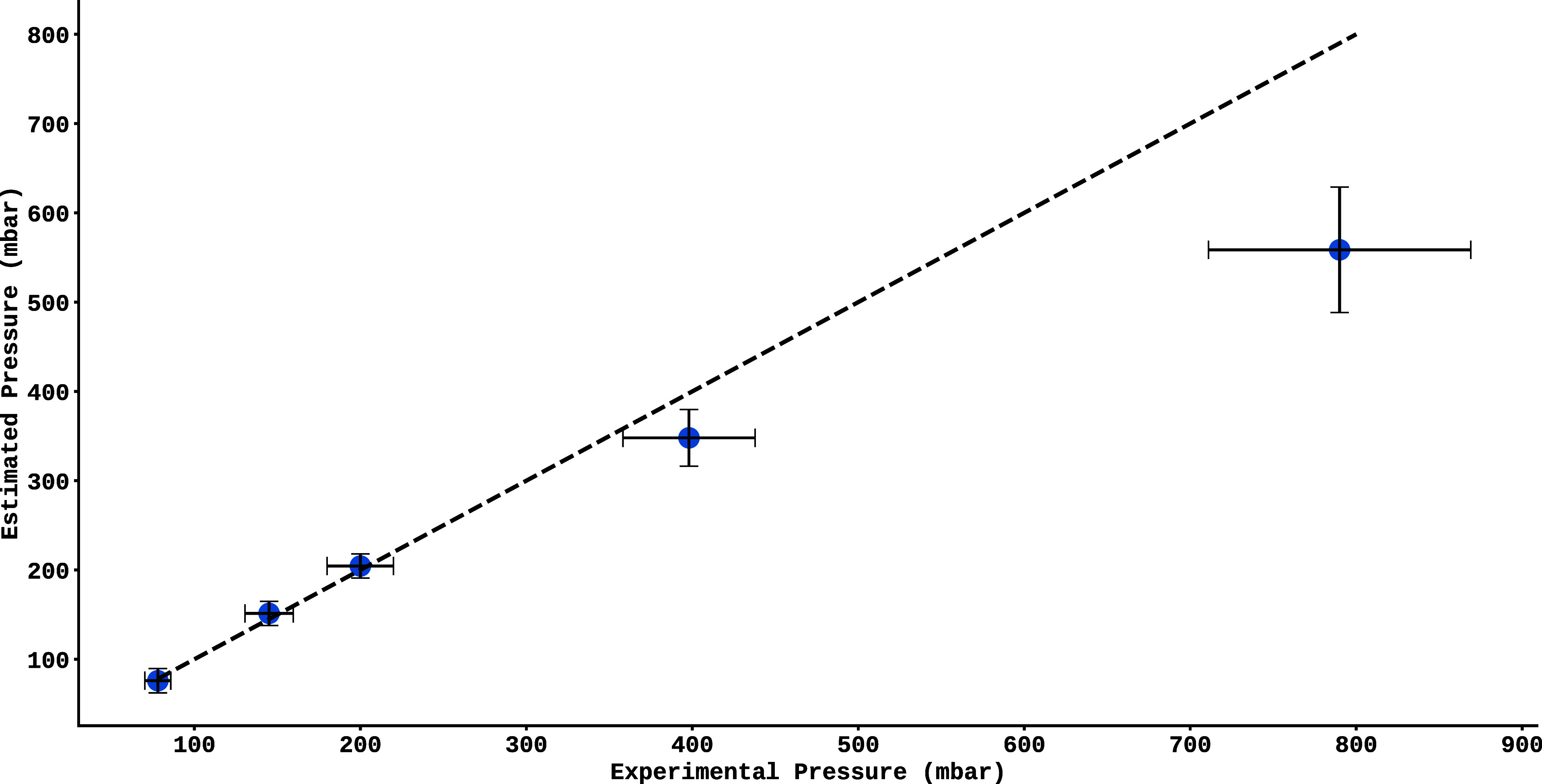
Validation of the Extra-Trees model on experimental spectra, comparing the pressure estimated by the algorithm (*y*-axis) with the real values measured by physical detectors (*x*-axis). The error associated with the estimated values was obtained by calculating the variance of the estimated values for each corresponding pressure, as explained in the text.

In Figure 9, we also present the error intervals associated with the experimental pressures (*x*-axis) and the model estimates (*y*-axis). On the *x*-axis, the error bars reflect the uncertainties associated with the experimental measurements of HCl pressure. Defining uncertainty for model predictions is a distinct challenge, as machine learning model outputs are generally point-specific. To overcome this limitation, we performed a statistical analysis of the residuals in order to establish representative error intervals. To do this, we used the information in Figure 8, which shows the residuals as a function of simulated pressure. As the residuals exhibit heteroscedasticity, our strategy was to divide the pressures into smaller intervals so that in each range it was possible to obtain residuals closer to homoscedasticity. The intervals defined were [0, 200], [200, 400], [400, 600], and [600, 800]. At each interval, we applied two different approaches to estimate the uncertainty of the model. The first consisted of calculating the standard deviation of the residuals twice, since, assuming that within each range they follow approximately a normal distribution, this value corresponds to a confidence interval of about 95*%*, since approximately 95*%*, of the residuals are expected to be contained within ±2 standard deviations. The second approach was to directly calculate the 95*%* confidence interval from the percentiles of the residual distribution, adopting the 2.5*%* percentile as the lower limit and the 97.5*%* percentile as the upper limit. This strategy, unlike the previous one, is nonparametric and therefore does not assume any specific form of distribution. Its main advantage is its robustness in the face of asymmetric distributions, but, on the other hand, it can result in nonsymmetric intervals, in addition to being less common in certain applications. When comparing the intervals obtained by the two methods, we found that, although not identical, they were similar. Given this, we chose to adopt the criterion of ±2 standard deviations in each pressure interval, as it is a simpler and easier to interpret approach, without compromising the consistency of the results.

## Conclusions

In the present study it is proposed a noninvasive method for pressure estimation in gaseous systems from high-resolution HCl spectra, combining simulations based on the HITRAN database with machine learning techniques. The hybrid approach, experimentally validated, demonstrated a robust accuracy at moderate pressures (absolute errors of 2–6 mbar, 2–4% below 200 mbar), but showed increasing challenges in high pressure regimes (errors of 12–29% above 398 mbar), associated with spectral broadening and line overlap effects. The ExtraTrees model, optimized with a maximum depth of 17, a minimum split of 17 samples per node, and 362 estimators, stood out for its generalization between synthetic and experimental data, although underestimation trends at high pressures suggest limitations in the representation of nonlinear relationships or in the density of extreme data.

The developed methodology, centered on Voigt profile parameters (amplitude, Gaussian and Lorentzian widths) and tree ensembles, is directly transferable to other molecules of astrochemical and industrial interest, such as HF, HBr and CO, whose spectra share pressure broadening characteristics and distinct vibrational structures. Adaptation only requires re-evaluation of experimental conditions (e.g., pressure ranges, isotopologues) and adjustment of hyperparameters, while maintaining the feature extraction and cross-validation pipeline. In addition, the technique developed can also be applied to low-resolution spectra, further enhancing its range of application. As a next step, we intend to expand our data set by acquiring additional real spectra not only for HCl, but also for other important molecules such as those mentioned previously, obtained under different conditions of temperature, optical paths and pressure, to enrich the data set and increase the effectiveness of the model training.
